# The Need for Oral Frailty Prevention and Future Tasks

**DOI:** 10.56786/PHWR.2026.19.41suppl.3

**Published:** 2026-10-29

**Authors:** Nam-Hee Kim, Sun-Jung Shin, Hee-Jung Park, Min-A Kim, Jun-Hyuk Choi, Ae-Jung Im, Ji-Hye Lee, Jin-Young Jeong, Jung-Nam Jin, Chan-Young Park

**Affiliations:** 1 Department of Dental Hygiene, Mirae Campus, Yonsei University Wonju Korea; 2 Department of Dental Hygiene, College of Dentistry, Kangwon National University Gangneung Korea; 3 Department of Dental Hygiene, College of Health Science, Kangwon National University Samcheok Korea; 4 Department of Dental Hygiene, Kyungdong University Wonju Korea; 5 Department of Dental Hygiene, Catholic Kwandong University Gangneung Korea; 6 Department of Dental Hygiene, Hallym Sacred Heart University Chuncheon Korea; 7 Welfare and Health Bureau, Gangwon State Government Chuncheon Korea; 8 Hallym Research Institute of Clinical Epidemiology, Hallym University Chuncheon Korea; 9 Hoengseong-gun Public Health Center, Hoengseong Korea; 10 Department of Dental Hygiene, Graduate School, Yonsei University Wonju Korea

**Keywords:** Frailty, Oral health, Aged, Mass screening, Community health services

## Abstract

**Objectives**: To review the concept of oral frailty and the available evidence and propose priorities for community-based prevention and management in the Republic of Korea (ROK).

**Methods**: This narrative review examined studies and government and public agency reports on definitions, screening and diagnosis, epidemiological characteristics, interventions, and policy linkages. Findings were organized into four themes.

**Results**: Oral frailty is a potentially reversible intermediate state between normal oral function and oral hypofunction. It is associated with physical frailty, sarcopenia, and mortality and affects approximately one-third of community-dwelling older adults in pooled international estimates. It may precede physical frailty, can be identified using self-reported questions, and may provide information on nutritional pathways that conventional frailty assessments do not adequately capture. In the ROK, self-reported screening items have been proposed, but their reliability when administered by community personnel remains unestablished, while confirmatory assessment relies on specialized devices. Korean intervention evidence consists mainly of small, single-site pre-post studies and case reports. This review identified no Korean empirical studies demonstrating that oral frailty interventions improve physical frailty. Linkages between local health programs and national oral health policy remain at the pilot stage.

**Conclusions**: Four priorities are proposed: incorporate oral frailty screening and assessment into community frailty assessment and link identified needs to nutrition and muscle-strengthening care; integrate and standardize existing self-reported items, evaluate inter-rater agreement, test-retest reliability, and field acceptability among non-specialists, and refer only those at risk for professional assessment; adapt internationally supported interventions to the ROK delivery system and assess oral function, frailty, nutrition, and muscle strength in multicenter studies; and link local health programs with national policy through referral pathways and screening arrangements.

**Key messages**

**① What is known previously?**

Oral frailty is associated with physical frailty, sarcopenia, and mortality; Korean cohort data also link chewing or speaking difficulties with incident frailty.

**② What new information is presented?**

In the Republic of Korea (ROK), confirmatory oral frailty assessment requires specialized devices, while the reliability of self-reported screening items administered by community personnel remains unestablished. This review identified no Korean studies demonstrating that oral frailty interventions improve physical frailty. Linkage between local health programs and national policy remains at the pilot stage.

**③ What are implications?**

Priorities are to integrate oral frailty assessment with community frailty assessment and nutrition and muscle-strengthening care; standardize and validate existing self-reported items for first-stage screening; adapt and evaluate internationally supported interventions within the ROK delivery system; and link local health programs with national health policy.

## Introduction

The “Act on the Integrated Support for Community-Based Medical, Long-Term Care, and Related Services,” which came into effect in March 2026, requires local governments to provide medical, long-term care, and related services in an integrated manner [1]. Integrated care must first ensure that people in need of care receive sufficient services to meet their needs. However, given the finite availability of care personnel and financial resources, as well as the need to maintain individuals’ ability to perform activities of daily living and their quality of life, a preventive approach that enables the early detection and management of functional decline before care needs arise should be implemented in parallel. The World Health Organization’s Integrated Care for Older People (ICOPE) approach assesses six domains of intrinsic capacity—cognition, locomotion, vitality (including nutrition), vision, hearing, and psychological capacity—and recommends assessment and early care planning in primary care [2].

According to the Ministry of Health and Welfare’s 2023 Survey of Older Persons, based on the Korean version of the FRAIL scale (K-FRAIL), 4.6% of older adults in the Republic of Korea (ROK) were frail and 32.2% were in a state of pre-frailty. Based on these findings, the Korea Disease Control and Prevention Agency (KDCA) has highlighted the policy need for frailty prevention and is pursuing the development of a Korean frailty prevention program model, standardized frailty prevention models for urban, urban-rural mixed, and rural settings, and monitoring of frailty status through the Community Health Survey [3]. However, discussions of frailty prevention have largely focused on physical function, falls, and declining muscle strength, whereas the pathway through which declining oral function may lead to physical frailty has received comparatively little attention.

Oral frailty refers to a reversible intermediate state between a healthy oral condition and clinically diagnosed oral hypofunction. The concept has been developed academically since the Japan Geriatrics Society, Japanese Society of Gerodontology, and Japanese Association on Sarcopenia and Frailty formed a joint consortium in 2014 [4,5]. The Japanese Kashiwa cohort study followed 2,011 adults aged ≥65 years and found that participants with oral frailty, defined as impairment in at least three of six items and observed in 16% of the cohort, subsequently had significantly increased risks of physical frailty, sarcopenia, disability, and mortality by 2.4-, 2.2-, 2.3-, and 2.2-fold, respectively. These findings support the possibility that oral frailty is not merely an oral health problem but a risk factor that precedes physical frailty [6]. In the ROK, several studies using nationally representative data, including the Korea National Health and Nutrition Examination Survey (KNHANES), have reported associations of chewing difficulty and fewer remaining teeth with frailty [7,8]. More recently, a six-year follow-up study using the Korean Frailty and Aging Cohort Study (KFACS) found that self-reported difficulty chewing or speaking increased the risk of incident frailty (adjusted hazard ratio [HR], 1.53; 95% confidence interval [CI], 1.19–1.98) and the risk of transitioning from a healthy state to pre-frailty (HR, 1.26; 95% CI, 1.09–1.47), while reducing the likelihood of recovery (HR, 0.78; 95% CI, 0.66–0.92). These findings further support the possibility that declining oral function is an independent predictor of frailty among older adults in the ROK [9].

Despite this evidence, strategies for integrating oral frailty into community-based frailty management systems in the ROK remain at an early stage. Therefore, this study aimed to summarize the conceptual and epidemiological evidence on oral frailty and to propose four future priorities that must be addressed for community implementation: ① incorporating oral frailty screening and assessment into community frailty assessment procedures and linking identified needs to nutrition and muscle-strengthening interventions; ② integrating and standardizing existing self-reported screening items into a single first-line community screening tool and evaluating its reliability when used by non-specialists and its acceptability in practice; ③ adapting and evaluating intervention components derived from international evidence to the ROK service delivery system; and ④ establishing linkages between local (oral) health programs and national (oral) health policy.

## Methods

This study was conducted as a narrative literature review. We reviewed professional society consensus statements and clinical practice guidelines addressing the definition and assessment criteria of oral frailty; cohort studies and systematic reviews reporting prevalence and health outcomes; clinical trials and systematic reviews of oral function interventions; and legislation, government reports, program guidelines, and national statistics from the ROK and Japan. The reviewed evidence was synthesized into four themes: ① the association between oral frailty and physical frailty, ② screening and diagnostic tools, ③ intervention programs, and ④ policy linkages. No separate systematic search and selection process or meta-analysis was conducted, and quantitative values were derived from the cited literature.

## Results

### 1. Association between Oral Frailty and Physical Frailty

The policy relevance of oral frailty lies in its position along a pathway wherein declining oral function may lead to physical frailty through reduced nutritional intake and subsequent declines in muscle strength and physical function. The World Health Organization’s ICOPE guidelines identify six domains for intrinsic capacity screening—cognition, locomotor capacity, vitality (including nutrition), vision, hearing, and psychological capacity—but oral function has not yet been included as an independent screening domain [2]. However, the vitality-domain screening items on unintentional weight loss and appetite loss may be related to impaired chewing and swallowing, leaving scope for oral function assessment to be incorporated into this pathway. A meta-analysis of community-dwelling older adults reported a pooled prevalence of oral frailty of 32% (95% CI, 24–40%) [10]. Another meta-analysis that did not restrict the residential setting reported a prevalence of 34.0% (95% CI, 27.9–40.1%), including 32.1% among community-dwelling older adults [11], while the consensus statement jointly issued by three Japanese academic societies estimated the prevalence at approximately 40% [4]. These differences are attributable to variation in the definitions and assessment tools applied. Taken together, the pooled estimates suggest that oral frailty is common among older adults, although prevalence varies substantially by definition and setting.

Evidence is also accumulating regarding associations among oral frailty, sarcopenia, and malnutrition. In the ROK, a policy research report by the National Evidence–based Healthcare Collaborating Agency found, through a propensity score-matched cross-sectional analysis of KNHANES data, that the prevalence of physical frailty was significantly higher among individuals with oral functional limitations than among those without such limitations (51.0% vs. 42.6%). The KFACS also showed that lean body mass and serum protein concentrations differed significantly according to the presence of oral functional limitations and the number of remaining teeth [12]. Internationally, a prospective cohort study that followed 51 patients undergoing peritoneal dialysis (mean age, 59.1 years) for one year found that the oral frailty group had significantly poorer trajectories than the non-oral-frailty group in terms of physical frailty, skeletal muscle index, calf circumference, and indicators of nutritional status [13]. However, because this study had a small sample and involved a specific patient population, its findings cannot be directly generalized to community-dwelling older adults. A 12-week cluster randomized controlled trial of an oral frailty improvement program in Japan, which randomized 30 dental clinics and analyzed 83 participants, found significant improvements in masticatory ability, oral diadochokinesis, and tongue pressure, demonstrating that oral function interventions can alleviate oral frailty. The authors also recommended further research to verify subsequent effects on the prevention of physical frailty and improvement of nutritional status [14].

The studies in the ROK described above, together with community-based longitudinal and screening-tool studies from Japan, have indicated that oral frailty screening and assessment possess three characteristics that justify their inclusion as components of community frailty assessment procedures. First is temporal precedence. In longitudinal studies conducted both in the ROK and internationally, declining oral function has preceded and predicted incident physical frailty [6,9]. Second is ease of assessment. Chewing, swallowing, and articulation can be assessed using self-reported questions or simple observation, unlike muscle strength testing or gait-speed measurement [4,15]. Third is the provision of additional information. Conventional frailty assessment items—weight loss, muscle strength, gait speed, physical activity, and fatigue—do not identify why nutritional intake has declined; oral function items may help identify chewing or swallowing difficulties that contribute to reduced intake [12]. For example, administering questions on chewing, swallowing, and articulation to individuals classified as pre-frail may identify those whose weight loss is attributable to difficulty chewing, allowing oral function exercises and nutritional counseling to be provided together. Conversely, the absence of problems in oral function may indicate the need to investigate other causes.

On the basis of these three characteristics, oral frailty screening and assessment should be treated not as optional additions to frailty assessment procedures, but as components that operate within those procedures. However, for this structure to function in practice, both the applicability of screening tools and the gaps in the intervention evidence discussed below must be addressed.

### 2. Current Status of Oral Frailty Screening and Diagnostic Tools and the Need for Adaptation for Use by Non-specialists

Most internationally used oral frailty screening tools were developed with community-based application by non-specialists in mind. The Oral Frailty Index-8 (OFI-8) consists of eight self-reported items and scores oral function, behavior, and social activity from 0 to 11 without requiring specialized dental equipment. It has been evaluated for its ability to identify risk of incident oral frailty and functional disability [15]. Similarly, the oral frailty five-item checklist (OF-5) proposed in the consensus statement jointly issued by three Japanese academic societies explicitly emphasizes that assessment can be performed without dental professionals [4]. However, the initial OF-5 used in the 9-year follow-up of the Kashiwa cohort included objective assessments of tooth count and articulatory oral motor skills. This follow-up demonstrated its predictive validity, showing that older adults with an OF-5 score of ≥2 had significantly increased risks of physical disability (adjusted HR, 1.40; 95% CI, 1.14–1.72) and mortality (adjusted HR, 1.44; 95% CI, 1.11–1.87) [16].

However, oral frailty diagnostic criteria developed in the ROK rely heavily on dental clinical measurement tools and equipment. The proposed Korean diagnostic criteria for oral frailty developed by the National Evidence–based Healthcare Collaborating Agency through a review of literature from the ROK and other countries and a Delphi survey of 26 experts define oral frailty as the presence of positive findings in at least three of the following six categories: masticatory ability, occlusal force, swallowing function, salivary gland function, oral cleanliness, and tongue-lip motor function. Many of these items consist of objective clinical tests requiring specialized measurement tools or devices, including color-changing chewing gum, Dental Prescale II^®^, and MUCUS^®^. Impaired masticatory ability must be included among the three positive items [12]. Similarly, a study applying the 2022 oral frailty diagnostic criteria proposed by the Korean Academy of Geriatric Dentistry (KAGD) to 206 adults aged ≥60 years in Wonju, Gangwon Province, assessed five of the six items using specialized devices, including color-changing chewing gum, Dental Prescale II^®^, a JMS tongue pressure meter, and MUCUS^®^ [17]. A study of 100 participants compared the screening questionnaire and diagnostic criteria proposed by the KAGD with the Tanaka criteria. The questionnaire had a sensitivity of 87.8% and specificity of 52.5%, while the diagnostic criteria had a sensitivity of 95.1% and specificity of 42.4%. The authors considered both instruments useful for identifying oral frailty; the low specificity nevertheless calls for careful interpretation of positive results. For community implementation, a staged approach can begin with self-reported screening and refer those at risk for professional assessment [18]. A Korean clinical practice guideline for oral frailty similarly recommends a staged approach in recognition of the practical difficulty of conducting detailed diagnostic testing for all individuals. It recommends first administering simple screening questions that can be completed by patients, family caregivers, or care workers, followed by referral for professional diagnosis only when screening identifies risk. At the time of its publication, the guideline noted that a standardized oral frailty assessment tool tailored to conditions in the ROK had not yet been established [19]. Reflecting the same concern, the National Evidence–based Healthcare Collaborating Agency report also proposed a 12-item self-reported Oral Frailty Risk Screening Questionnaire and recommended a two-step process wherein adults aged ≥50 years undergo screening, and diagnostic testing is conducted only among those assessed as being at risk or at high risk [12].

Taken together, self-reported screening items and a two-step assessment process have already been proposed in the ROK. The remaining gap concerns validation for routine use by nondental community personnel and evidence on field feasibility. The proposed screening instruments differ in the number of items and scoring methods, inter-rater agreement and test-retest reliability when administered by community-based personnel have not been established, and evidence from actual public health center settings remains limited. Therefore, the priority for future work is not to replace confirmatory criteria based on specialized measurement tools, but to standardize the existing screening items into a single tool and validate it in community settings. Specifically, existing self-reported tools such as the OF-5, OFI-8, and KAGD screening questionnaire should be compared and adapted to community conditions in the ROK, revised using language that is easy for older adults to understand, and evaluated for feasibility when administered by public health center staff, home-visiting health care personnel, and community health facilitators. Through this process, standardized oral frailty screening items should be incorporated into the first-line screening stage, with only individuals identified as being at risk of oral frailty referred for professional diagnostic assessment.

### 3. Current Status of Evidence-based Intervention Programs and the Need for Application and Validation in the ROK

Although the international evidence base for oral frailty interventions has grown quantitatively, its certainty remains low. A meta-analysis of 11 randomized controlled trials involving 734 participants found that oral exercise interventions significantly improved oral diadochokinesis (/pa/), tongue pressure, masticatory function, and maximum occlusal force [20]. A 2026 systematic review synthesized 19 studies qualitatively and included eight in its meta-analysis. Benefits were reported for swallowing function after tongue exercises and for masticatory function after masticatory exercises; lip exercises did not significantly improve swallowing function [21]. A systematic review of 23 studies involving 2,412 participants aged ≥65 years, evaluated using the Grading of Recommendations Assessment, Development and Evaluation framework, found the most consistently favorable directions of effect for oral motor outcomes such as tongue pressure and oral diadochokinesis in studies of oral exercises alone. Multicomponent programs combining oral exercises with physical activity or nutrition showed positive trends across several domains, including overall oral health status, although the evidence was limited because few studies were available. Overall certainty of evidence ranged from very low to low, underscoring the urgent need for standardized intervention protocols [22]. Similarly, a systematic review examining associations between oral health interventions and sarcopenia or frailty concluded that oral exercise and swallowing interventions may have the potential to improve frailty- and sarcopenia-related indicators; however, substantial heterogeneity and low certainty of evidence warrant large-scale, standardized clinical trials [23].

Of the eight studies included in the systematic review examining the effects of oral health interventions on sarcopenia and frailty [23], seven were conducted in Japan and one in Finland, demonstrating substantial geographic concentration of the evidence. Furthermore, systematic reviews have consistently identified heterogeneity in intervention protocols and outcome measures and low certainty of evidence [20-23]. Evidence from the ROK remains limited to small-scale studies conducted in single regions. In one eight-week pre-post study of oral exercises and tongue-strengthening exercises among 14 adults aged ≥65 years at a local public health center, the results of oral mucosal moisture, orbicularis oris muscle strength, and repetitive saliva swallowing tests improved, whereas subjective symptoms and tongue pressure did not; the study also had limited representativeness [24]. A case study of home-visiting oral health care in Cheonan also reported improvement in halitosis, but included only three participants, and one participant showed deterioration relative to baseline at 12 weeks, resulting in a low level of evidence [25].

Particularly notable is the finding from the National Evidence–based Healthcare Collaborating Agency’s policy research that, after reviewing 19,285 publications from the ROK and other countries, the report identified no study from the ROK evaluating physical frailty outcomes after an oral frailty intervention [12]. Although oral function may improve after intervention, whether such improvements prevent physical frailty remains to be tested in the ROK.

Therefore, core intervention components centered on oral exercises that have shown benefits in Japan and other countries may be prioritized for use in pilot programs in the ROK [14,20-23]. However, whereas Japan has operated linkage through health checkups for the old-old and community comprehensive support centers, as well as an intervention study based in 30 dental clinics [14,26,27], the settings where such interventions would be applied in the ROK are the frailty prevention and management pilot program and the home-visiting oral health care pilot program, which are run by different departments within public health centers, and there is no formal linkage with the national health screening [28-30]. Furthermore, in a study validating the KAGD diagnostic criteria against the Tanaka criteria among 100 community-dwelling older adults in the ROK, the specificity of the KAGD criteria was 42.4% [18]. This single validation study suggests that the criteria may classify individuals differently and that their performance should be examined in the target population before intervention protocols are transferred across settings. Therefore, the core components of international interventions should be adopted, but the responsible providers, implementation settings, and assessment criteria should be adapted to the ROK service delivery system, and their feasibility and effectiveness should be evaluated through multicenter pragmatic studies. Measuring the frailty phenotype, Mini Nutritional Assessment, and handgrip strength concurrently would help clarify the intervention’s contribution to the prevention of physical frailty [12,13].

### 4. Linkage between Local (Oral) Health Programs and National (Oral) Health Policy

Japan has already integrated oral function assessment into its national health screening system. The 15-item Questionnaire for Medical Checkup of Old-Old for adults aged ≥75 years consists of ten domains and includes two independent oral function items addressing difficulty in chewing and choking [26]. A study on health insurance claims data from 24,836 individuals in Japan found that participants classified as frail based on the total questionnaire score had 1.28-fold higher medical expenditures (95% CI, 1.18–1.39) and 3.47-fold higher long-term care expenditures (95% CI, 2.62–4.60), demonstrating an association between the overall screening score, which includes oral function items, and health care expenditures [31]. In addition, a research support project of Japan’s Ministry of Health, Labour and Welfare developed a community implementation manual for oral frailty measures based on the OF-5 checklist for use by local government administrative personnel and relevant professionals [32]. The Japanese model introduced by the FDI World Dental Federation also describes a structure wherein community comprehensive support centers serve as coordination hubs linking dental institutions, medical institutions, and long-term care facilities, together with a procedure for referring individuals who screen positive on oral function items during frailty screening to community services [27].

The ROK is at an early stage of developing such an integrated system. In the second basic plan for oral health programs issued in 2022, the Ministry of Health and Welfare stated that it would consider introducing masticatory function testing tailored to the characteristics of adults and older adults [28]. In 2025, a pilot home-visiting oral health care program was implemented in 29 cities, counties, and districts nationwide, under which dentists and dental hygienists visited homebound older adults with mobility limitations to provide oral hygiene assessments and oral function training [29]. In 2026, a pilot frailty prevention and management program was launched at eight public health centers and two Healthy Living Support Centers—three in urban areas, four in urban-rural mixed areas, and three in rural or fishing areas. Multidisciplinary teams comprising exercise specialists, dietitians, dental hygienists, and other professionals conduct frailty screening assessments among adults aged ≥65 years and provide integrated management encompassing exercise, nutrition, and oral health. The program is planned to expand to 60 sites in 2027, 100 sites in 2028, and all 264 sites nationwide by 2030 [30]. The frailty prevention and management pilot program is significant because it is a national pilot program in the ROK wherein oral health has been formally incorporated as a component of an integrated frailty prevention and management model. However, it remains at the pilot stage, and no formal linkage has yet been established with either national health screening or national oral health screening. Moreover, the pilot home-visiting oral health care program is overseen by the Ministry of Health and Welfare’s Division of Oral Health Policy, whereas the frailty prevention and management pilot program is overseen by the Ministry’s Health Policy Bureau, and KDCA is separately pursuing the standardization of frailty definitions and assessment systems. Therefore, because program guidelines and documentation forms are operated separately, an explicit linkage structure connecting these initiatives needs to be established.

The need for such linkage is also demonstrated by regional disparities. Although the data sources differ, KDCA’s 2024 Community Health Survey found that the prevalence of chewing difficulty among adults aged ≥65 years had a median of 27.7% across cities, counties, and districts, with values ranging widely from 12.6% to 41.0% [33]. In KNHANES, the prevalence of oral functional limitations among adults aged ≥70 years, defined as difficulty chewing or speaking, was 39.2% in 2022. This prevalence remained high in contrast to the improvement observed among the overall population aged ≥19 years, for whom the corresponding prevalence declined from 20.1% in 2013 to 14.8% in 2022 [34]. The Dental Health Act establishes the responsibilities of the national and local governments to formulate and implement oral health program plans and provides the legal basis for conducting oral health status surveys [35]. Thus, discussion could begin on incorporating oral frailty screening and intervention into local oral health programs and national oral health screening. KDCA also stated in its 2026 work plan that it would establish standardized national definitions and assessment systems for frailty and monitor frailty status at the provincial and city/county/district levels to support the development of tailored strategies [3]. Therefore, consideration should be given to incorporating oral frailty screening and linkage procedures into this standardized framework.

## Discussion

Evidence from the ROK and other countries indicates that oral frailty is associated with increased risks of physical frailty, sarcopenia, and mortality and has been reported as a predictor of these outcomes [6,9]. In pooled international studies, approximately one-third of community-dwelling older adults met the respective criteria for oral frailty [10,11]. However, the accumulation of evidence is not equivalent to the successful implementation of that evidence in community settings. The delayed implementation of oral frailty prevention in the ROK is not due to an absence of evidence, but rather to the lack of clearly established arrangements regarding who should identify oral frailty, which tools should be used, what evidence should guide interventions, and where individuals should be referred. To address these issues, four priorities should be considered (Table 1).

First, oral frailty screening and assessment should be incorporated as components of community frailty assessment procedures. Prospective studies suggest that declining oral function may precede physical frailty. The former can be identified through self-reported questions or simple observation and may provide clues about nutritional pathways underrepresented in conventional frailty assessment items. Linking this assessment to nutritional counseling and necessary dental treatment could support not only oral function but also the maintenance of nutritional status and muscle strength.

Second, existing self-reported screening items should be integrated and standardized into a single first-line community screening tool. Item wording and number, assessment thresholds, and training procedures for screeners should be established. Inter-rater agreement, test-retest reliability, and acceptability in practice should then be evaluated when the tool is administered by non-specialists, such as public health center staff, home-visiting health care personnel, and community health facilitators. A two-step structure should subsequently be established wherein only individuals identified as being at risk are referred for professional diagnostic assessment.

Third, core intervention components centered on oral exercises that have demonstrated oral function benefits in Japan and other countries should be prioritized for use in pilot programs in the ROK while accounting for differences between the dental clinic–centered intervention environment in Japan and the public health center– and home-visiting care–centered service delivery system and assessment criteria in the ROK [14,18,29,30]. Multicenter pragmatic studies conducted alongside implementation should establish standardized intervention protocols and core outcome measures and evaluate feasibility and effectiveness by assessing frailty indicators, nutritional status, and muscle strength together with oral function.

Fourth, linkage mechanisms should be established between local (oral) health programs and national (oral) health policy. Specifically, community oral health programs and frailty prevention and management programs implemented by public health centers should be connected within the policy guidelines of the Ministry of Health and Welfare and KDCA; referral and back-referral pathways to primary health care institutions and dental care institutions should be defined; and the inclusion of oral function assessment in national health screening and national oral health screening should be considered. Drawing on Japan’s experience with integration into national health screening, the outcomes of the ROK frailty prevention and management pilot program and home-visiting oral health care program should inform a standardized national frailty prevention program manual to reduce barriers to coordination and improve acceptability and sustainability in practice [3,30].

In the ROK, which has entered a super-aged society, the prevention of oral frailty can no longer be solely considered an oral health issue. Rather, it should be addressed as a shared policy agenda within an integrated frailty prevention and management system. When evidence-generating research addressing these four priorities is pursued in parallel with interagency collaboration, oral frailty management may become a key component of the national frailty prevention policy.

## Figures and Tables

**Table 1:** Four tasks for applying oral frailty prevention in Korean communities

Task	Key activities	Deliverables
① Integration with frailty assessment	Include oral frailty items in frailty screening; link positive results to nutrition counseling, muscle-strengthening care, and dental treatment through standardized pathways and forms	Screening-intervention pathway; record form
② Integration and standardization of a community first-stage screening tool	Integrate existing self-report items into a first-stage screening tool and assess their reliability and acceptability among nondental personnel	Integrated tool; administration guide; validation
③ Local adoption and evaluation of evidence-based interventions	Adapt international core components to the Republic of Korea’s delivery system; assess feasibility and effectiveness in multicenter studies	Standard intervention; core outcomes; multicenter evaluation
④ Linkage of local programs and national policy	Link referral and records across oral health and frailty prevention programs; consider oral function in national screening	Referral system; options for national screening; disparity indicators
