# Supplementary material for: The Need for Oral Frailty Prevention and Future Tasks
Source: Public Health Wkly Rep. 2026 Oct 29;19(41 Suppl):37–58. doi: 10.56786/PHWR.2026.19.41suppl.3 (PMC13724136; doi:10.56786/PHWR.2026.19.41suppl.3)
Supplement: Translation

# Translation: 구강노쇠 예방의 필요성과 향후 과제

**Authors:** 남희 김, 선정 신, 희정 박, 민아 김, 준혁 최, 애정 임, 지혜 이, 진영 정, 정남 진, 찬영 박

**Affiliations:** 1 연세대학교 미래캠퍼스 치위생학과; 2 강원대학교 치과대학 치위생학과; 3 강원대학교 보건과학대학 치위생학과; 4 경동대학교 치위생학과; 5 가톨릭관동대학교 치위생학과; 6 한림성심대학교 치위생과; 7 강원특별자치도 복지보건국; 8 한림대학교 사회의학연구소; 9 횡성군보건소; 10 연세대학교 일반대학원 치위생학과

**Keywords:** 노쇠, 구강건강, 노인, 선별검사, 지역사회보건서비스

## Abstract

**목적:**

이 논문은 구강노쇠(oral frailty)의 학술적 개념과 국내외 근거를 고찰하고, 국내 지역사회 기반의 예방‧관리 체계 구축을 위한 전략적 과제를 제안하고자 하였다.

**방법:**

이 논문은 서술적 문헌고찰로 수행하였다. 구강노쇠의 개념과 정의, 선별‧진단 도구, 역학적 특성, 중재 효과, 국내외 정책 연계에 관한 학술문헌과 정부‧공공기관 보고서를 검토하고 네 가지 주제로 종합하였다.

**결과:**

구강노쇠는 정상 구강기능과 임상적 구강기능저하증(oral hypofunction) 간의 가역적 중간 상태로, 신체적 노쇠, 근감소증, 사망률 증가와 유의하게 연관되며 지역사회 노인 인구의 약 3분의 1에서 확인된다. 구강노쇠는 신체노쇠에 선행하며, 자가보고식 설문으로 식별 가능하고, 기존 노쇠 척도가 반영하지 못하는 영양 매개 경로를 보완한다는 점에서 통합적 노쇠 평가 체계의 구성요소로서 타당성을 지닌다. 국내에서도 자가보고형 선별문항이 제안되어 있으나, 확진 단계의 진단 기준은 전용 측정 기기에 의존하며 지역사회 인력이 선별문항을 적용했을 때의 신뢰도는 아직 검증되지 않았다. 또한 중재 근거는 단일 기관 대상의 소규모 전후비교 연구 및 증례보고에 편중되어 있으며, 구강노쇠 중재가 신체노쇠 개선에 미치는 영향을 검증한 국내 실증 연구는 찾아볼 수 없었다. 아울러 지역보건사업과 국가 단위 구강보건정책 간의 연계 구조 역시 시범사업 수준에 정체되어 있다.

**결론:**

구강노쇠 예방을 지역사회에 적용하기 위해 첫째, 구강노쇠 선별‧판정을 노쇠 평가 절차 안에서 작동하게 하여 영양‧근력 중재로 연결한다. 둘째, 기존 자가보고형 선별문항을 하나의 지역사회 1차 선별도구로 통합‧표준화하고, 비전문가가 시행했을 때의 검사자 간 일치도‧재검사 신뢰도‧현장 수용성을 검증한 뒤 위험군만 전문가 진단으로 연계하는 2단계 구조를 정착시킨다. 셋째, 국외에서 효과가 보고된 중재요소를 국내 시범사업에 우선 적용하되 국내 전달체계에 맞게 조정하고, 구강기능‧노쇠‧영양상태‧근력을 평가하는 다기관 연구로 실행가능성과 효과를 검증한다. 넷째, 지역(구강)보건사업과 국가(구강)보건정책을 연계하여 의뢰‧회송과 검진 경로를 제도화해야 한다.

**핵심요약**

**① 이전에 알려진 내용은?**

구강노쇠는 신체노쇠‧근감소증‧사망 위험을 높이는 독립적 위험요인으로 보고되었으며, 국내 연구에서도 저작 곤란과 구강기능 저하가 노쇠 위험 증가와 일관되게 연관되었다.

**② 새로이 알게 된 내용은?**

국내 구강노쇠 확진 기준은 전용 측정 기기를 요구하고, 선별문항은 지역사회 인력이 시행했을 때의 신뢰도가 검증되지 않았으며, 구강노쇠 중재가 신체노쇠에 미치는 효과를 확인한 국내 연구는 없고, 지역보건사업과 국가보건정책의 연계도 시범사업 수준에 머물러 있다.

**③ 시사점은?**

구강노쇠 선별‧판정을 노쇠 평가 절차의 구성요소로 포함하여 영양‧근력 관리로 연결하고, 기존 자가보고 문항을 통합한 지역사회 1차 선별도구를 표준화‧검증하며, 국외 근거에 기반한 중재를 국내 전달체계에 맞게 적용‧평가하고, 지역(구강)보건사업과 국가(구강)보건정책을 연계하는 것이 향후 과제이다.

## 서 론

2026년 3월 시행된 「의료‧요양 등 지역 돌봄의 통합지원에 관한 법률」은 지방자치단체가 의료‧요양‧돌봄 서비스를 통합하여 제공하도록 하였다[1]. 통합돌봄은 우선 돌봄이 필요한 사람에게 필요한 서비스를 충분히 제공해야 한다. 하지만 돌봄 인력과 재정은 유한하고 개인의 일상생활 수행능력과 삶의 질을 유지해야 한다는 점을 고려하면, 돌봄이 필요해지기 전 기능 저하를 조기에 발견‧관리하는 예방 접근이 병행되어야 한다. 세계보건기구는 노인 통합관리(Integrated Care for Older People, ICOPE) 지침에서 질병으로 진단되기 전에 인지‧이동‧영양‧시력‧청력‧우울 증상 등 여섯 영역의 기능이 서서히 떨어지는 이 시기를 내재역량(intrinsic capacity)의 저하로 규정하고, 일차보건의료 수준에서 정기적으로 선별하여 조기에 개입할 것을 권고하였다[2].

보건복지부의 2023년 노인실태조사에 따르면 한국형 노쇠척도(K-FRAIL) 기준으로 국내 노인의 4.6%가 노쇠, 32.2%가 전노쇠(pre-frailty) 상태였다. 질병관리청은 이 결과를 근거로 노쇠 예방의 정책적 필요성을 제기하고, 한국형 노쇠예방 프로그램 모형 개발과 도시‧도농복합‧농촌형 노쇠예방 표준 모형 개발, 지역사회건강조사를 통한 노쇠 현황 모니터링을 추진하고 있다[3]. 그러나 노쇠 예방 논의는 대체로 신체기능, 낙상, 근력 저하에 초점이 맞춰져 있으며, 구강기능 저하가 신체노쇠로 이어지는 경로는 상대적으로 주목받지 못하고 있다.

구강노쇠(oral frailty)는 건강한 구강과 임상적으로 진단되는 구강기능저하증(oral hypofunction) 사이에 위치하는 가역적인 중간 상태를 의미하며, 2014년 일본노년의학회‧일본노년치의학회‧일본사르코페니아프레일학회가 협의체를 구성한 이후 학술적으로 정립해 왔다[4,5]. 일본의 가시와(Kashiwa) 코호트 연구는 65세 이상 2,011명을 추적하여, 6개 항목 중 3개 이상이 저하된 구강노쇠 해당자(16%)에서 이후 신체노쇠, 근감소증, 장애, 사망 위험이 각각 2.4배, 2.2배, 2.3배, 2.2배 유의하게 증가함을 보임으로써, 구강노쇠가 단순한 구강건강 문제가 아니라 신체노쇠에 선행하는 위험요인일 가능성을 뒷받침하였다[6]. 국내에서도 국민건강영양조사(Korea National Health and Nutrition Examination Survey, KNHANES) 등 전국 단위 자료를 이용한 여러 연구에서 저작 곤란과 잔존치아 수 감소가 노쇠와 유의하게 연관됨을 일관되게 보고하고 있으며[7,8], 최근 한국노인노쇠코호트(Korean Frailty and Aging Cohort Study, KFACS)를 6년간 추적한 연구는 자가보고 저작 또는 발음 불편이 노쇠 발생 위험을 높이고(보정 위험비, 1.53; 95% 신뢰구간, 1.19–1.98), 건강 상태에서 전노쇠 단계로의 이행 위험을 높이며(위험비, 1.26; 95% 신뢰구간, 1.09–1.47) 회복 가능성은 낮춘다는 것을(위험비, 0.78; 95% 신뢰구간, 0.66–0.92) 확인하여, 구강기능 저하가 국내 노인에서도 노쇠의 독립적 예측인자일 가능성을 뒷받침하였다[9].

이러한 근거에도 불구하고, 국내에서 구강노쇠를 지역사회 노쇠관리체계에 통합하기 위한 실행 전략은 아직 초기 단계에 머물러 있다. 이에 이 논문은 구강노쇠의 개념적‧역학적 근거를 정리하고, 지역사회 적용을 위해 해결해야 할 네 가지 향후 과제인 ① 구강노쇠 선별‧판정을 지역사회 노쇠 평가 절차의 구성요소로 포함하여 영양‧근력 중재로 연결하는 방안, ② 기존 자가보고형 선별문항을 하나의 지역사회 1차 선별도구로 통합‧표준화하고 비전문가의 사용 신뢰도와 현장 수용성을 검증하는 방안, ③ 국외 근거에 기반한 중재요소를 국내 전달체계에 맞게 적용‧평가하는 방안, ④ 지역(구강)보건사업과 국가(구강)보건정책의 연계 방안을 제시하는 것을 목적으로 하였다.

## 방 법

이 논문은 서술적 문헌고찰로 수행하였다. 구강노쇠의 정의‧판정기준을 다루는 학회 합의문(consensus statement)‧진료지침, 유병률‧건강결과를 보고한 코호트 연구‧체계적 문헌고찰, 구강기능 중재 임상시험‧체계적 문헌고찰, 한국‧일본의 법령‧정부 보고서‧사업 지침‧국가통계를 검토하였다. 검토한 근거를 ① 구강노쇠와 신체노쇠의 연관성, ② 선별‧진단 도구, ③ 중재 프로그램, ④ 정책 연계의 네 가지 주제로 종합하였다. 별도의 체계적 검색‧선정이나 메타분석은 수행하지 않았으며, 정량 수치는 인용 문헌에 보고된 수치를 사용하였다.

## 결 과

### 1. 구강노쇠와 신체노쇠의 연관성

구강노쇠 개념이 정책적으로 중요한 이유는, 구강기능 저하가 영양섭취 감소와 근력‧신체기능 저하를 매개하여 신체노쇠로 진행하는 경로상에 위치하기 때문이다. 세계보건기구의 ICOPE 지침은 내재역량 선별 영역으로 인지, 이동, 영양, 시력, 청력, 정서 여섯 가지를 제시하고 있으며, 구강기능은 아직 독립 선별 영역으로 포함되어 있지 않다[2]. 다만 영양 영역의 의도하지 않은 체중감소와 식욕저하 문항은 저작‧연하 기능 저하와 직접 맞닿아 있어, 구강기능 평가를 이 경로에 연결할 여지가 있다. 지역사회 거주 노인을 대상으로 한 메타분석에서 구강노쇠의 통합 유병률은 32% (95% 신뢰구간, 24–40%)로[10], 거주 형태를 제한하지 않은 메타분석에서는 34.0% (95% 신뢰구간, 27.9–40.1%)로 보고되었으며(이 중 지역사회 거주자는 32.1%) [11], 일본노년의학회 등 3개 학회가 공동으로 발표한 합의문은 약 40%로 제시하였다[4]. 수치의 차이는 적용한 정의와 판정도구가 다르기 때문이며, 어느 추정치를 따르더라도 노쇠 예방 대상 인구집단의 3분의 1 안팎이 구강노쇠를 동반한다는 점은 공통적이다.

구강노쇠와 근감소증‧영양불량의 연관성을 보여주는 근거도 축적되고 있다. 국내에서는 한국보건의료연구원의 정책연구 보고서가 KNHANES 자료의 성향점수매칭 단면분석을 통해 구강기능제한이 있는 군의 신체노쇠 유병률이 51.0%로 없는 군(42.6%)보다 유의하게 높고, KFACS에서는 제지방질량(lean body mass)과 혈중 단백질 농도가 구강기능제한 여부 및 잔존치아 수에 따라 유의하게 달랐음을 확인하였다[12]. 국외에서도 복막투석 환자 51명(평균 59.1세)을 1년간 추적한 전향적 코호트에서 구강노쇠군이 신체노쇠, 골격근지수, 종아리둘레, 영양상태 지표 모두에서 비구강노쇠군보다 유의하게 나쁜 경과를 보였는데[13], 이는 표본이 작고 특정 환자군을 대상으로 한 결과이므로 지역사회 노인에게 그대로 적용하기는 어렵다. 일본에서 30개 치과의원을 무작위 배정하여 83명을 분석한 12주 구강노쇠 개선 프로그램 클러스터 무작위대조시험은 저작능력, 조음교대운동, 설압이 유의하게 향상되어 구강기능 중재가 구강노쇠를 완화시킬 수 있음을 보였으며, 이후 신체노쇠 예방 및 영양상태 개선 효과에 대한 후속 검증이 필요함을 제언하였다[14].

앞서 제시한 국내 연구와 일본의 지역사회 기반 종단연구 및 선별도구 연구는 구강노쇠 선별‧판정이 지역사회 노쇠 평가 절차의 구성요소로 포함될 만한 세 가지 성질을 갖추고 있음을 보여준다. 첫째, 선행성이다. 구강기능 저하는 신체노쇠에 앞서 나타나며 국내외 종단연구에서 노쇠 발생을 유의하게 예측하였다[6,9]. 둘째, 측정의 용이성이다. 저작‧연하‧조음 기능은 근력검사나 보행속도 측정과 달리 자가보고 문항이나 간단한 관찰만으로도 파악할 수 있다[4,15]. 셋째, 추가 정보의 제공이다. 체중감소‧근력‧보행속도‧활동량‧피로로 구성된 기존 노쇠 평가 항목은 영양섭취가 왜 줄었는지를 설명하지 못하지만, 구강기능 문항은 그 원인을 짚어 중재로 연결할 수 있는 정보를 더해 준다[12]. 예를 들어 전노쇠로 분류된 사람에게 저작‧연하‧조음 문항을 이어서 적용하면, 체중이 줄어든 이유가 씹기 어려움에 있는 사람을 가려내어 구강기능 운동과 영양상담을 함께 제공할 수 있고, 구강 문항에서 문제가 없다면 다른 원인을 찾아야 한다는 신호가 된다.

이 세 가지 성질을 근거로, 구강노쇠 선별과 판정은 노쇠 평가 절차에 덧붙이는 선택 항목이 아니라 그 안에서 함께 작동하는 구성요소로 다룰 필요가 있다. 다만 이 구조가 실제로 작동하려면 아래에서 다루는 선별도구의 적용성 문제와 중재 근거의 공백이 함께 해결되어야 한다.

### 2. 구강노쇠 선별‧진단 도구의 현황과 비전문가 적용을 위한 조정 필요성

국제적으로 통용되는 구강노쇠 선별도구는 대부분 비전문가의 지역사회 적용을 염두에 두고 개발되었다. 자가보고 8문항으로 구성된 Oral Frailty Index-8 (OFI-8)은 치과 전문 장비 없이 구강기능‧행동‧사회활동 관련 항목을 0–11점으로 점수화하여 신규 구강노쇠와 기능장애 위험을 예측하도록 설계하였다[15]. 일본노년의학회 등 3개 학회가 공동으로 발표한 합의문이 제시한 5문항 자가체크리스트(oral frailty five-item checklist, OF-5) 역시 치과 전문인력 없이도 평가할 수 있다는 점을 명시적으로 강조하였다[4]. 다만 가시와 코호트의 9년 추적연구에는 치아 수와 조음교대운동에 대한 객관적 평가를 포함한 초기 OF-5가 사용되었다. 이 연구에서 OF-5 2점 이상인 노인은 신체장애(보정 위험비, 1.40; 95% 신뢰구간, 1.14–1.72)와 사망(보정 위험비, 1.44; 95% 신뢰구간, 1.11–1.87) 위험이 유의하게 증가하여 예측타당도가 확인되었다[16].

그러나 국내에서 개발된 구강노쇠 진단기준은 치과임상 측정도구와 장비에 대한 의존도가 높다. 한국보건의료연구원이 국내외 문헌고찰과 26인 전문가 델파이 조사를 거쳐 제시한 한국형 구강노쇠 진단기준(안)은 저작능력, 교합력, 삼킴기능, 타액선기능, 구강청결, 설구순운동능력 등 6개 범주 중 3개 이상 양성 시 구강노쇠로 진단하도록 하였는데, 이들 항목의 상당수는 색변화 저작검사용 껌, Dental Prescale II^®^, MUCUS^®^ 등 전용 측정 도구나 기기를 요구하는 객관적 임상검사로 구성되어 있으며, 3개 항목 중 저작능력 저하는 반드시 포함하도록 하였다[12]. 강원도 원주 지역 60세 이상 206명에게 대한노년치의학회(Korean Academy of Geriatric Dentistry, KAGD)가 2022년 제시한 구강노쇠 진단기준을 적용한 연구에서도 6개 항목 중 5개를 시행하면서 색변화 저작검사용 껌, Dental Prescale II^®^, JMS 설압계, MUCUS^®^ 등 전용 기기를 사용하였다[17]. Tanaka 기준을 참조표준으로 삼아 KAGD의 선별문항과 진단기준을 검증한 국내 연구(대상자 100명)는 두 도구 모두 민감도는 높으나(각각 87.8%, 95.1%) 특이도는 낮다는 점(각각 52.5%, 42.4%)을 보여주었다. 따라서 조기 선별에 활용할 수 있으나 양성 결과는 신중히 해석하고 추가 평가할 필요가 있다. 지역사회에서는 자가보고 선별을 먼저 시행하고 위험군을 전문가 평가로 연계하는 2단계 접근을 고려할 수 있다[18]. 대한치과의사협회의 구강노쇠 임상진료지침 역시 전체 대상자에게 정밀 진단검사를 시행하기 어려운 현실을 고려하여, 환자‧보호자‧간병인이 작성 가능한 간이 선별문항을 1단계로 우선 시행하고 선별문항에서 위험으로 분류된 경우에만 전문가 진단으로 연계하는 단계적 접근을 권고하면서, 우리나라 실정에 맞는 표준화된 구강노쇠 평가도구가 아직 개발되어 있지 않다는 점도 함께 지적하였다[19]. 한국보건의료연구원의 보고서 역시 이러한 문제의식에 따라 12문항 자가보고 「구강노쇠 위험도 선별 문진표」를 함께 제시하고, 50세 이상에게 선별검사를 적용하여 위험 또는 고위험으로 평가된 경우에만 진단검사를 시행하는 2단계 절차를 권고하였다[12].

종합하면, 자가보고형 선별문항과 2단계 절차라는 설계는 국내에서도 이미 제시되어 있다. 남은 공백은 도구의 부재가 아니라 검증의 부재다. 제안된 선별문항들은 문항 수와 배점이 서로 다르고, 지역사회 인력이 시행했을 때의 검사자 간 일치도와 재검사 신뢰도가 확인된 바 없으며, 실제 보건소 현장에 적용한 연구도 보고되지 않았다. 따라서 향후 과제는 전용 측정 도구에 기반한 확진 기준 자체를 대체하는 것이 아니라, 이미 제안된 선별문항을 하나로 표준화하고 지역사회 현장에서 검증하는 데 있다. 구체적으로는 OF-5, OFI-8 및 KAGD 선별문진표처럼 이미 제안된 자가보고형 문항을 한국 지역사회 여건에 맞게 재구성하고, 고령자가 이해하기 쉬운 표현으로 다듬으며, 보건소 담당자‧방문건강관리 인력‧건강활동가가 시행했을 때의 적용가능성 등을 확인하는 절차가 필요하다. 이 과정을 거쳐 표준 구강노쇠 선별문항을 1차 선별 단계에 배치하고, 구강노쇠 위험군으로 분류된 사람만 전문 진단으로 연계하는 구조를 정착시켜야 한다.

### 3. 근거 기반 중재 프로그램의 현황과 국내 적용‧검증의 필요성

구강노쇠 중재에 관한 국제 근거는 양적으로는 축적되었으나 근거의 확실성은 아직 낮은 수준에 머물러 있다. 11개 무작위대조시험(참가자 734명)을 종합한 메타분석은 구강운동 중재가 구강교대운동(/pa/), 설압, 저작기능, 최대교합력을 유의하게 개선함을 확인하였다[20]. 2026년 체계적 문헌고찰은 19편을 정성적으로 종합하고 8편을 메타분석하였다. 설운동은 연하기능을, 저작운동은 저작기능을 개선하는 결과를 보였으나, 구순운동의 연하기능 개선 효과는 통계적으로 유의하지 않았다[21]. 65세 이상을 대상으로 한 23개 연구(참가자 2,412명)를 근거수준 평가체계(Grading of Recommendations Assessment, Development and Evaluation, GRADE)로 평가한 체계적 문헌고찰은 구강운동 단독 중재가 설압과 구강교대운동 등 운동기능에서 가장 일관된 개선을 보였고, 다중요소(구강운동에 신체활동 또는 영양을 결합) 프로그램은 종합적 구강건강상태를 포함한 여러 영역에서 긍정적 방향성을 보였으나 포함된 연구 수가 적어 근거가 제한적이라고 보고하였다. 근거의 확실성은 전반적으로 매우 낮음(very low)에서 낮음(low) 수준에 머물러 표준화된 중재 프로토콜 정립이 시급하다[22]. 구강건강 중재와 근감소증‧노쇠의 연관성을 검토한 체계적 문헌고찰 역시 구강운동과 연하 중재가 노쇠‧근감소증 관련 지표를 완화할 잠재적 역할을 할 수 있으나, 이질성이 크고 근거의 확실성이 낮아 대규모 표준화 임상시험이 필요하다고 결론지었다[23].

구강건강 중재가 근감소증‧노쇠에 미치는 영향을 검토한 체계적 문헌고찰[23]의 8편 중 7편은 일본, 나머지 1편은 핀란드에서 수행되어 근거의 지역적 편중이 확인된다. 또한 체계적 문헌고찰들은 중재 프로토콜과 결과지표의 이질성 및 낮은 근거 확실성을 공통으로 지적한다[20-23]. 국내 근거는 단일 지역의 소규모 연구에 머물러 있다. 일부 지역 보건소의 65세 이상 노인 14명을 대상으로 한 8주 입체조‧설근력 강화 전후비교 연구에서는 구강점막 수분력, 구륜근력, 반복타액삼킴검사가 개선되었으나 자각증상과 설압 차이는 없었고 대표성이 제한된다[24]. 천안시 방문 구강건강관리 사례연구도 구취 개선을 보고했으나 대상이 3명이고 1명은 12주에 기저치보다 악화되어 근거 수준이 낮다[25].

특히 주목할 점은 한국보건의료연구원의 정책연구가 국내외 문헌 19,285편을 검토한 결과 구강노쇠 중재와 신체노쇠의 관련성을 평가한 국내 문헌이 한 편도 없었다는 사실이다[12]. 구강노쇠 중재가 구강기능을 개선한다는 근거는 있으나, 그 개선이 신체노쇠 예방으로 이어지는지를 확인한 국내 연구는 아직 없다.

일본 등 국외 연구에서 효과가 보고된 구강운동 중심의 핵심 중재요소는 국내 시범사업에 우선 적용할 수 있다[14,20-23]. 다만 일본은 후기고령자 검진‧지역포괄지원센터를 통한 연계와 30개 치과의원 기반 중재연구를 운영한 반면[14,26,27], 한국의 적용 현장은 서로 다른 부서가 운영하는 보건소 노쇠예방관리‧방문 구강건강관리 시범사업이며 국가검진과의 공식 연계가 없다[28-30]. 또한 Tanaka 기준을 준거로 한국 지역사회 노인 100명에게 검증한 연구에서 KAGD 진단기준의 특이도는 42.4%였다[18]. 두 판정기준으로 분류되는 대상자가 다를 수 있으므로, 국외 중재를 국내에 적용하기 전에 대상 집단에서 기준의 성능을 확인할 필요가 있다. 따라서 국외 중재의 핵심요소는 도입하되, 시행 주체‧장소‧판정기준은 국내 전달체계에 맞게 조정하고 다기관 실용적 연구로 실행가능성과 효과를 검증해야 한다. 이때 노쇠 표현형(frailty phenotype), 간이영양상태조사(Mini Nutritional Assessment), 악력을 함께 측정하면 신체노쇠 예방 기여도를 확인할 수 있다[12,13].

### 4. 지역(구강)보건사업과 국가(구강)보건정책의 연계

일본은 구강기능 평가를 국가 단위 건강검진체계에 이미 통합하였다. 후기고령자(75세 이상)용 15문항 건강검진 문진표(Questionnaire for Medical Checkup of Old-Old, QMCOO)는 10개 영역으로 구성되며, 구강기능 영역에 저작 곤란과 사레 2문항을 독립 배치한다[26]. 일본 건강보험 청구자료 24,836명을 분석한 연구는 이 문진표 총점으로 노쇠에 해당한다고 판정된 사람의 의료비가 1.28배(95% 신뢰구간, 1.18–1.39), 장기요양비가 3.47배(95% 신뢰구간, 2.62–4.60) 높았음을 보여, 구강기능 문항을 포함한 검진 총점이 의료비 지출과 관련됨을 확인하였다[31]. 아울러 일본 후생노동성 연구지원사업은 OF-5 체크리스트를 기반으로 지자체 행정직원과 관련 전문직이 활용할 수 있는 지역 실천형 구강노쇠 대책 매뉴얼을 개발하였다[32]. 국제치과연맹(FDI World Dental Federation)이 소개한 일본 모델은 지역포괄지원센터가 치과‧의료기관‧장기요양시설을 조정하는 허브로 기능하는 구조와, 노쇠 선별 시 구강기능 문항에서 양성일 경우 지역사회서비스로 연계하는 절차를 함께 제시한다[27].

한국은 이러한 통합체계 구축의 초기 단계에 있다. 보건복지부는 2022년 제2차 구강보건사업 기본계획에서 성인‧노인 특성을 고려한 저작기능검사 도입을 검토하겠다고 밝혔으며[28], 2025년에는 거동이 불편한 재가노인을 대상으로 치과의사‧치과위생사가 가정을 방문해 구강위생평가와 구강기능훈련을 제공하는 방문 구강건강관리 시범사업을 전국 29개 시‧군‧구에서 시행하였다[29]. 2026년에는 보건소 8개소와 건강생활지원센터 2개소(도시형 3, 도농복합형 4, 농어촌형 3)에서 운동전문가‧영양사‧치과위생사 등으로 구성된 다학제 팀이 65세 이상을 노쇠 선별평가한 뒤 운동‧영양‧구강건강을 통합 관리하는 노쇠예방관리 시범사업이 시작되었으며, 2027년 60개소, 2028년 100개소를 거쳐 2030년까지 전국 264개소로 확대할 계획이다[30]. 노쇠예방관리 시범사업은 한국에서 구강건강이 노쇠예방 통합관리 모델의 정식 구성요소로 포함된 국가 시범사업이라는 점에서 중요하다. 다만 아직 시범사업 단계에 머물러 있고, 국가건강검진 및 국가구강검진과의 공식적 연계 체계도 마련되어 있지 않다. 또한 방문 구강건강관리 시범사업은 보건복지부 구강정책과가, 노쇠예방관리 시범사업은 보건복지부 건강정책국이 주관하며, 질병관리청은 노쇠의 정의‧평가체계 표준화를 별도로 추진하고 있어 사업 지침과 기록 서식이 분리되어 운영되므로, 이들을 잇는 연계 구조를 명시적으로 정리할 필요가 있다.

이러한 연계 구축의 필요성은 지역 간 격차 자료에서도 확인된다. 자료원이 서로 다르기는 하나, 질병관리청의 2024년 지역사회건강조사에 따르면 65세 이상 저작불편호소율의 전국 시‧군‧구 중앙값은 27.7%였으며 시‧군‧구 단위로는 최저 12.6%에서 최고 41.0%까지 큰 편차를 보였다[33]. KNHANES에서 저작 또는 발음 불편으로 정의한 70세 이상의 구강기능제한율은 2022년 39.2%로, 19세 이상 전체가 2013년 20.1%에서 2022년 14.8%로 개선된 것과 대조적으로 여전히 높은 수준을 유지하고 있다[34]. 구강보건법은 국가와 지방자치단체에 구강보건사업 계획 수립‧시행 의무와 구강건강실태조사 실시 근거를 부여하고 있으므로[35], 이를 토대로 구강노쇠 선별‧중재를 지역구강보건사업과 국가구강건강검진 항목에 포함하는 논의를 시작할 수 있다. 질병관리청 역시 2026년 업무계획에서 노쇠 개념과 평가체계를 국가 표준으로 정립하고 시‧도 및 시‧군‧구별 노쇠 현황을 모니터링하여 맞춤형 전략 수립을 지원하겠다고 밝힌 바 있어[3], 이 표준 체계에 구강노쇠 선별과 연계 절차를 포함하는 방안을 검토할 필요가 있다.

## 논 의

구강노쇠는 국내외 근거에서 신체노쇠‧근감소증‧사망 위험 증가와 관련되며 이를 예측하는 요인으로 보고되었고[6,9], 지역사회 노인의 약 3분의 1이 이에 해당한다[10,11]. 그러나 근거가 쌓였다는 것과 그 근거가 지역사회에서 작동한다는 것은 다른 문제다. 국내에서 구강노쇠 예방이 지연되는 이유는 근거가 없어서가 아니라, 누가 어떤 도구로 찾아내고 무엇을 근거로 중재하며 어디로 연계할지가 정해지지 않았기 때문이다. 이를 풀기 위해 다음 네 가지 과제를 함께 고려할 필요가 있다(표 1).

첫째, 구강노쇠 선별과 판정을 지역사회 노쇠 평가 절차의 구성요소로 포함할 필요가 있다. 구강기능 저하는 신체노쇠에 선행하고, 자가보고 문항이나 간단한 관찰만으로도 파악할 수 있으며, 기존 노쇠 평가 항목이 설명하지 못하는 영양 경로의 정보를 더해 준다. 이를 영양상담과 필요한 치과치료로 연계하면 구강기능뿐 아니라 영양상태와 근력 보존을 함께 지원할 수 있다.

둘째, 기존 자가보고형 선별문항을 하나의 지역사회 1차 선별도구로 통합‧표준화할 필요가 있다. 문항 표현과 개수, 판정 기준, 시행자 훈련 절차를 정하고, 보건소 담당자‧방문건강관리 인력‧건강활동가 등 비전문가가 시행했을 때의 검사자 간 일치도‧재검사 신뢰도‧현장 수용성을 검증한 뒤, 위험군만 전문가 진단으로 연계하는 2단계 구조를 정착시켜야 한다.

셋째, 일본 등 국외에서 효과가 보고된 구강운동 중심의 핵심 중재요소는 국내 시범사업에 우선 적용하되, 치과의원 중심의 일본 중재환경과 보건소‧방문관리 중심의 국내 전달체계 및 판정기준의 차이를 반영해야 한다[14,18,29,30]. 적용과 병행한 다기관 실용적 연구에서는 표준 중재 프로토콜과 핵심결과지표를 정하고, 구강기능과 함께 노쇠 지표‧영양상태‧근력을 평가하여 실행가능성과 효과를 검증해야 한다.

넷째, 지역(구강)보건사업과 국가(구강)보건정책을 잇는 연계 방안을 마련할 필요가 있다. 구체적으로는 보건소가 수행하는 지역사회 구강보건사업과 노쇠예방관리사업을 보건복지부‧질병관리청의 정책 지침 안에서 연결하고, 일차보건의료기관과 치과의료기관으로 이어지는 의뢰‧회송 경로를 정하며, 국가건강검진과 국가구강검진 항목에 구강기능 평가를 포함하는 방안을 검토하는 것이다. 일본의 국가 단위 검진 통합 사례를 참고하여 국내 노쇠예방관리 시범사업과 방문 구강건강관리 사업의 성과를 국가 표준 노쇠예방사업 매뉴얼에 반영함으로써 연계 장벽을 줄이고 현장 수용성과 지속성을 높인다[3,30].

초고령사회에 접어든 한국에서 구강노쇠 예방은 더 이상 구강건강 영역에 국한된 과제가 아니라, 노쇠 예방의 통합적 관리체계 안에서 다루어져야 할 공동의 정책 의제이다. 위 네 가지 과제에 대한 근거창출 연구와 다부처 협력이 병행될 때, 구강노쇠 관리는 국민 노쇠 예방정책의 실질적인 구성요소로 자리매김할 수 있을 것으로 기대한다.

## Figures and Tables

**표 1:** 지역사회 구강노쇠 예방 적용을 위한 네 가지 과제

과제	핵심 내용	산출물
① 노쇠 평가 절차와의 결합	노쇠 선별 절차에 구강노쇠 문항 포함; 양성자를 영양상담‧근력관리‧치과치료로 연결; 절차‧서식 표준화	선별‧중재 연계 절차, 기록 서식
② 지역사회 1차 선별도구의 통합‧표준화	기존 자가보고 문항을 하나의 1차 선별도구로 통합; 비전문가 사용 신뢰도‧수용성 검증	통합 선별도구, 시행 지침, 검증 결과
③ 국외 근거 기반 중재의 국내 적용‧검증	국외 핵심 중재요소를 국내 전달체계에 맞춰 적용; 다기관 연구로 실행가능성‧효과 평가	표준 중재안, 핵심결과지표, 다기관 평가
④ 지역사업과 국가정책의 연계	구강보건‧노쇠예방 사업의 의뢰‧회송‧기록 연계; 국가검진 구강기능 평가 검토	의뢰‧회송 체계, 검진 개정안, 격차 지표
